# The cost-effectiveness of point of care testing in a general practice setting: results from a randomised controlled trial

**DOI:** 10.1186/1472-6963-10-165

**Published:** 2010-06-15

**Authors:** Caroline O Laurence, John R Moss, Nancy E Briggs, Justin J Beilby

**Affiliations:** 1 Discipline of General Practice, School of Population Health and Clinical Practice, The University of Adelaide, North Terrace, Adelaide, South Australia, Australia; 2 Discipline of Public Health, School of Population Health and Clinical Practice, The University of Adelaide, North Terrace, Adelaide, South Australia, Australia; 3 Data Management & Analysis Centre, Discipline of Public Health, School of Population Health and Clinical Practice, The University of Adelaide, North Terrace, Adelaide, South Australia, Australia; 4 Faculty of Health Sciences, The University of Adelaide, North Terrace, Adelaide, South Australia, Australia

## Abstract

**Background:**

While point of care testing (PoCT) for general practitioners is becoming increasingly popular, few studies have investigated whether it represents value for money. This study aims to assess the relative cost-effectiveness of PoCT in general practice (GP) compared to usual testing practice through a pathology laboratory.

**Methods:**

A cost-effectiveness analysis based on a randomized controlled trial with 4,968 patients followed up for 18 months and fifty-three general practices in urban, rural and remote locations across three states in Australia.

The incremental costs and health outcomes associated with a clinical strategy of PoCT for INR, HbA1c, lipids, and ACR were compared to those from pathology laboratory testing. Costs were expressed in year 2006 Australian dollars. Non-parametric bootstrapping was used to generate 95% confidence intervals.

**Results:**

The point estimate of the total direct costs per patient to the health care sector for PoCT was less for ACR than for pathology laboratory testing, but greater for INR, HbA1c and Lipids, although none of these differences was statistically significant. PoCT led to significant cost savings to patients and their families. When uncertainty around the point estimates was taken into account, the incremental cost-effectiveness ratio (ICER) for PoCT was found to be unfavourable for INR, but somewhat favourable for ACR, while substantial uncertainty still surrounds PoCT for HbA1c and Lipids.

**Conclusions:**

The decision whether to fund PoCT will depend on the price society is willing to pay for achievement of the non-standard intermediate outcome indicator.

**Trial registration:**

Australian New Zealand Clinical Trial Registry ACTRN12605000272695

## Background

While a number of studies have been undertaken on PoCT in a primary care setting, few of these have included an economic analysis. [1] Hobbs et al [2], in a systematic review of PoCT in primary care, were unable to draw a conclusion on its cost-effectiveness because of insufficient data. More recently, the National Academy of Clinical Biochemistry's systematic review of PoCT [3] and an updated systematic review of PoCT in GP [4] have confirmed the paucity of reliable evidence on the cost-effectiveness of PoCT. Some studies indicate that PoCT is more expensive when compared to laboratory testing [5], but it has been argued that the costs of rapid availability of results may be justified by long term societal gains such as prolonged life or by reduced hospital stays [1]. However, it should be noted that the cost-effectiveness of PoCT is likely to vary according to the disease and the test in question [5]. Since the overall resources available to the health system are inevitably limited, it is important to ensure that the resources allocated to these tests are optimised.

The cost-effectiveness analysis was conducted alongside the Point of Care Testing in General Practice Trial, a randomised control trial in Australia [6]. Four tests were assessed in the Trial - internationalised normalised ratio (INR) of the prothrombin time, glycated hemoglobin (HbA1c), lipids (total cholesterol, triglycerides and HDL-C) and urine albumin creatinine ratio (ACR). The aim of the present analysis was to assess the incremental cost-effectiveness of a clinical strategy based on performing PoCT in GP compared to current practice of testing through a pathology laboratory. The perspective was that of Australian society overall.

## Methods

### PoCT Trial

This study was part of the Point of Care Testing in General Practice Trial which was conducted in 2005-2007. It was an Australian Government funded multi-centre, cluster randomised controlled trial to determine the safety and clinical effectiveness of PoCT in general practice and stakeholder satisfaction with its use [6]. Briefly, practices were recruited from three geographic locations (urban, rural and remote) and from these practices patients were recruited with diabetes, hyperlipidaemia and/or being on anticoagulant therapy. A total of 4968 patients were recruited, with 3010 and 1958 patients in the intervention and control groups respectively. Patients in the intervention group had their samples taken in the practice, with pathology testing being performed using three PoCT devices located there. Patients in the control group had their samples taken either in the practice and forwarded to the local laboratories for pathology testing or at the local laboratory or collection centre. For both groups, there was no patient copayment for the pathology testing. The Trial has been described elsewhere, providing in detail the methodology, rationale, recruitment process and baseline patient characteristics. The latter are provided in Table 1[6]. Ethical approval was obtained from five independent Australian Human Research Ethics Committees.

**Table 1:** Summary characteristics of patients

	Characteristics Freq (%)	Intervention (n = 3010)	Control (n = 1958)	Total (n = 4968)
Sex	Male	1630 (54.2)	1018 (52.0)	2648 (53.3)
Age group (years)	18-39	52 (1.7)	21 (1.1)	73 (1.5)
	40-49	199 (9.6)	109 (5.6)	308 (6.2)
	50-59	573 (19.0)	335 (17.1)	908 (18.3)
	60-69	1015 (33.7)	604 (30.8)	1619 (32.6)
	70-79	867 (28.8)	680 (34.7)	1547 (31.1)
	80+	304 (10.1)	209 (10.7)	513 (10.3)
	Age (years) median (IQ range)	66.0 (59.0-74.0)	68.0 (60.0-75.0)	67.0 (59.0-75.0)
Geographic region	Urban	897 (29.8)	840 (42.9)	1737 (35.0)
	Rural	917 (30.5)	447 (22.8)	1364 (27.5)
	Remote	1196 (39.7)	671 (34.3)	1867 (37.6)
Condition*	Anticoagulant therapy	572 (19.0)	372 (19.0)	944 (19.0)
	Diabetes	1182 (39.3)	785 (40.1)	1967 (39.6)
	Hyperlipidaemia	2356 (78.3)	1463 (74.7)	3819 (76.9)

*Patients could have more than one condition

### Resource use and Unit costs

Resource use data were collected prospectively during the Trial. The costs of the PoCT strategy were calculated by quantifying the resources used and assigning to them the relevant unit cost. Initial costs, induced costs and averted costs were considered. Fees and charges were used as proxies for opportunity costs. The actual point of care test costs were based on the Medicare Australia fees established for the Trial. The sources of unit cost and volume data for each type of test are outlined in Table 2. The cost analysis was undertaken using 18 months of Trial data. Costs were expressed in calendar year 2006 Australian dollars, adjusted where necessary by the Consumer Price Index (CPI) [7]. For comparison, over that year, one Australian dollar was worth 0.453 English pounds (based on purchasing power parities) [8].

**Table 2:** Resource use items and unit costs for all tests

Category of resource	Description	Source	Volume	Unit Cost
Establishment Program	PoCT equipment	Industry sources	Equivalent annual cost over clinically useful life (3 years)	
	Device Training initial and refresher	Trial	Twice - at commencement of Trial and 12 months later	
	Accreditation	Trial	Annual	

Pathology testing	Pathology tests - PoCT and laboratory testing*	Medicare Australia data	No. of tests claimed	100% of MBS fee for PoCT. 85% of schedule fee for laboratory test**
	Consultations - including where PoCT test ordered	Medicare Australia data	No. of consultations	Actual charge
	Copayments***	Medicare Australia data	No. of consultations	Actual charge minus MBS fee rebate
	Device Operator time (test, notes etc)	Time and motion study of sample of practices	No. of tests claimed	SA Nurses Award - Registered Nurse 3^rd ^year + oncosts
	Patient follow-up of test results (GP and nurse)	Time and motion study	No. of tests claimed	SA Nurses Award - Registered Nurse 3^rd ^year + oncosts SADI Division GP claims policy 2005-2007
	Patient Episode Initiation	Medicare Australia data	No.r of episodes claimed	85% of schedule fee**

Quality management	Quality Assurance Program	Industry source	Annual	
	Quality control	Device Group	No. of QC tests (monthly)	
	Quality assurance	RCPA QAP Pty Ltd	No. of QA tests (monthly	
	Device Operator time for QA and QC	Time and motion study	Number of QA and QC tests	SA Nurses Award - Registered Nurse 3^rd ^year + oncosts

Consumables and maintenance	Consumables - per test items and periodically used items****	Industry sources		Per test cost
	Annual maintenance fee			Nil. Manufacturers replaced defective devices at no cost.

Downstream costs	Hospital admissions (related only to disease group in study)	Case note audit on sample of patients	No. of visits	National Hospital Data Collection - Public Section Estimated Round 9 (2004-05) - AR-DRG 5.0*****
	Emergency department visits	Patient satisfaction survey	No. of visits	National Hospital Data Collection - Public Section Estimated Round 9 (2004-05) - AR-DRG 5.0
	Specialist referrals	Medicare Australia data	No. of referrals	MBS fee
	Allied health visits	Medicare Australia data	No. of referrals	MBS fee when referred by a GP
	Pharmaceutical costs	Medicare Australia data	No. of prescriptions for conditions associated with the test	PBS dispensed price and copayment

Patient costs	Motor vehicle travel	Patient satisfaction survey	Distance (Km)	Australian Taxation Office
	Other travel costs (eg bus, taxi)	Patient satisfaction survey	Mean cost	
	Time seeking healthcare (travel time, waiting time)	Patient satisfaction survey Time and motion study	Mean travel and waiting time	ABS seasonally adjusted average weekly earnings - applied whether were employed, unemployed or retired

* The effect of coning out of pathology was accounted for. Pathology providers can charge through the Medicare Benefits Schedule (MBS) fee only for the three most expensive tests ordered on the one occasion even when more tests are actually done. This is known as 'coning' and means that the tests recorded in the MBS data include only those charged for and not all that were done.

**87% of GP requested pathology is bulk-billed.

*** The high percentage of patients in the Trial who held health care cards or were pensioners (91%) and the location of practices in lower socio-economic status areas meant that most Medicare care items used in the Trial were bulked-billed so that patients did not make a copayment.

****Combined costs of items required to undertake each test. This included the testing strip/cassette, lancet, capillary tubes, plungers, urine pots, dipsticks and gloves. Periodically used items such as dust filters and cleaning kits were costed on an annual basis.

*****Hospitalisation rates were applied to a sample of patients obtained through the case note audit and weighted estimates generated for the Trial population to determine estimates for the whole sample. This approach was taken due to poor reporting of hospitalisations by practices. The hospitalisations were then assigned an AR-DRG code by a researcher blinded to the patient identification and their allocation to intervention or control under guidance from an expert AR-DRG coder. AR-DRG costs were adjusted by the CPI.

### Health outcome indicator

The intermediate outcome indicator used in the cost-effectiveness analysis was the proportion of patients within the therapeutic range for each condition at the end of the Trial [6,9]. The point estimates and confidence intervals for the health outcome indicators are presented in Bubner et al [9]. The short time horizon of the trial mitigated against using survival as an outcome indicator.

### Statistical analyses

In general, the strategy was to calculate the overall increment in cost for all practices and participants over the Trial period. Multiple imputation was used to address missing data issues.

Generalised Estimating Equations (GEE) were used to estimate the predicted number of healthcare item usages for a participant in the intervention and control arms, controlling for gender, age and correlation at the practice level. Estimates were adjusted for the length of time involved in the Trial. A Poisson model with log link was used to analyse count data; continuous data were assumed to be normally distributed.

From the original sample, 500 bootstrap samples were obtained, sampling at the practice level. This resulted in each bootstrap sample having different numbers of subjects (due to different numbers of subjects attending each practice), but over all bootstrap samples, the correlation due to clustering at the practice level was controlled. The 2.5th and 97.5th percentile values of the distribution obtained from bootstrapping the GEE estimates were taken as the limits of the 95% confidence intervals (CIs). CIs were obtained for unit costs and mean costs and differences.

All imputations and analyses were performed with SAS 9.1 (Cary, NC, USA).

### Sensitivity analysis

To examine the influence of uncertainty in the variables, one-way sensitivity analyses were undertaken on each resource item for which the underlying variable had a distribution. The upper and lower limits of the CIs were used as the ranges for the sensitivity analyses, which were only conducted on the health service costs, as the patient and family costs were of not sufficient magnitude to influence the overall results.

To represent uncertainty around cost and effect data, a joint distribution of these estimates was generated using non-parametric bootstrapping [10]. The resulting bootstrapped distributions of incremental cost and incremental effectiveness were plotted, along with the actually-observed base case.

## Results

### Cost comparisons

The point estimate of the total direct costs per patient to the health care sector for PoCT was less for ACR than pathology laboratory testing, but greater for INR, HbA1c and lipids, although none of these differences was statistically significant. The cost comparisons for HbA1c which illustrate the uncertainties involved are shown in Table 3, and for the other tests in Additional File 1.

**Table 3:** Comparison of costs (direct and indirect) at 18 months for HbA1c tests - costs per patient (Australian dollars, calendar year 2006)

RESOURCES	INTERVENTION (PoCT) N = 1182	CONTROL (Laboratory) N = 785	DIFFERENCE (INTERVENTION - CONTROL)
**Direct Costs to the health care sector**	**(95% CI)**	**(95% CI)**	**(95% CI)**
Establishment costs in GP	$114	$0*	$114
Consumables & maintenance in GP	$28 ($22, $31)	$0*	$28 ($22, $31)
Quality assurance & control in GP	$29	$0*	$29
HbA1c tests (100% MBS fee in GP, 85% MBS fee in pathology laboratory)	$64 ($49, $65)	$98 ($88, $107)	-$34 (-$54, -$28)

**Sub total cost of actual test**	**$235 ($184, $290)**	**$98 ($88, $107)**	**$137 ($84, $194)**
GP consultations	$579 ($515, $659)	$560 ($502, $618)	$18 (-$69, $112)
Hospital admissions	$171 (-$181, $662)	$506 (-$137, $1,241)	-$334 (-$1,131, $460)
Emergency Dept visits	$8 ($3, $13)	$8 ($2, $13)	-$1 (-$8, $7)
Specialist consultations	$173 ($163, $183)	$169 ($157, $180)	$4 (-$11, $20)
Allied health visits	$221 ($195, $253)	$232 ($188, $282)	-$11 (-$72, $47)
Pharmaceuticals	$2,249 ($1,889, $2,555)	$2,032 ($1,859, $2,586)	$217 (-$280, $253)
**Subtotal direct costs to healthcare sector**	**$3,636 ($3,019, $4,149)**	**$3,606 ($2,902, $4,563)**	**$30 (-$988, $673)**

**Direct Costs to the patients and families**			
Copayment for GP consultations and pharmaceuticals	$4 ($4, $6)	$4 ($2, $6)	$0 (-$2, $4)
Patient travel costs	$12 ($10, $14)	$28 ($23, $33)	-$16 (-$22, -$11)
**Subtotal direct costs to patients and families**	**$16 ($15, $19)**	**$32 ($28, $39)**	**-$16 (-$22, -$10)**

**Indirect Costs**			
**Time seeking healthcare**	**$23 ($22, $25)**	**$34 ($32, $37)**	**-$11 (-$14, -$8)**

**Total costs (both sectors)**			
**Total**	**$3,676 ($3,062, $4,191)**	**$3,672 ($2,972, $4,628)**	**$4 (-$1,103, $642)**

Note: totals not exact due to rounding

* For the control group these items are included in the MBS fee for the laboratory

Some relatively large differences were found in the cost components. Hospitalisation costs are reported below because of their magnitude, but other costs only where they were statistically significant. For HbA1c, PoCT resulted in reduced costs for hospital admissions and significantly increased costs for tests. For INR, PoCT resulted in reduced costs for hospital admissions and significantly reduced tests costs but significantly increased consultation costs. ACR by PoCT resulted in reduced costs for hospital admissions but significantly increased test costs. Lipids by PoCT resulted in increased hospitalisation and significantly increased pharmaceutical costs. For all tests, the intervention group had significantly lower patient costs for travel and time seeking healthcare.

### Incremental cost-effectiveness ratio point estimates

The effects per patient, the cost estimates and the point estimates of the ICER for PoCT compared to laboratory testing for each testing strategy are shown Table 4. ACR testing at the point of care dominates testing through a pathology laboratory, being both less costly and more effective. Conversely, INR by PoCT was dominated by its comparator (ie it was more expensive and less effective than laboratory testing). The point estimates of the ICERs for PoCT HbA1c and for Lipids fell in the north-east quadrant of the incremental cost-effectiveness plane, having higher costs and being more effective.

**Table 4:** Point estimates of the incremental cost-effectiveness ratios (ICER) for PoC testing per patient maintained in the target range* compared with laboratory testing

Test	Treatment Group	Costs per patient ($)	Effects per patient *	ICER ($)
INR	Intervention (PoCT)	3,298	0.5701	
	Control (Laboratory)	3,150	0.6147	
	Difference	148	-0.0446	Dominated

HbA1c	Intervention (PoCT)	3,676	0.6548	
	Control (Laboratory)	3672	0.5618	
	Difference	4	0.0930	$40

ACR	Intervention (PoCT)	1,727	0.7739	
	Control (Laboratory)	1,954	0.7418	
	Difference	-228	0.0321	Dominant

Lipids	Intervention (PoCT)	2,732	0.1592	
	Control (Laboratory)	2,202	0.1066	
	Difference	530	0.0526	$10,082

*Effect is the proportion of patients in target range as determined at the end of the Trial (18 months mean observation time)

### Sensitivity analyses

#### One-way sensitivity analyses of the incremental direct costs

The results of the one-way sensitivity analysis showed that, for all tests, the incremental costs per patient were most sensitive to differences in the costs of hospitalisation, and somewhat sensitive to differences in the costs of GP consultations, pharmaceuticals and allied health visits (see Additional Files 2).

#### One-way sensitivity analyses of the ICERs

The sensitivity of the base case ICER to changes in hospitalisations and GP consultations for each type of test is shown in Table 5. These one-way sensitivity analyses indicate that the ICER for each PoCT strategy is quite sensitive to variation in hospitalisation costs. Variations in GP costs have less influence, although the point estimate of the ICER for HbA1c is shifted into a different quadrant.

**Table 5:** One-way sensitivity analysis on the ICERs (as expressed in dollars per patient maintained in target range)

Test	Selected variables	Cost per patient maintained in target range
INR	Base ICER	Dominated*
	GP consultations - Upper 95% CI	Dominated*
	GP consultations - Lower 95% CI	Dominated*
	Hospital admissions - Upper 95% CI	Dominated*
	Hospital admissions - Lower 95% CI	SW Quadrant**

HbA1c	Base ICER	$40
	GP consultations - Upper 95% CI	$1,049
	GP consultations - Lower 95% CI	Dominant***
	Hospital admissions - Upper 95% CI	$8,579
	Hospital admissions - Lower 95% CI	Dominant***

ACR	Base ICER	Dominant***
	GP consultations - Upper 95% CI	Dominant***
	GP consultations - Lower 95% CI	Dominant***
	Hospital admissions - Upper 95% CI	$17,647
	Hospital admissions - Lower 95% CI	Dominant***

Lipids	Base ICER	$10,082
	GP consultations - Upper 95% CI	$11,691
	GP consultations - Lower 95% CI	$8,618
	Hospital admissions - Upper 95% CI	$26,120
	Hospital admissions - Lower 95% CI	Dominant***

* Dominated: PoCT was more costly and less effective than laboratory testing

** SW Quadrant: PoCT was less costly and less effective than laboratory testing

***Dominant: PoCT was less costly and more effective than laboratory testing

Note: this analysis was undertaken on all the other variables (consumables, tests, emergency department visits, specialist consultations and pharmaceuticals) but did not have an influence on the ICERs ie change the quadrant.

#### Joint probability distribution of the incremental costs and incremental effectiveness

**Figure 1:**
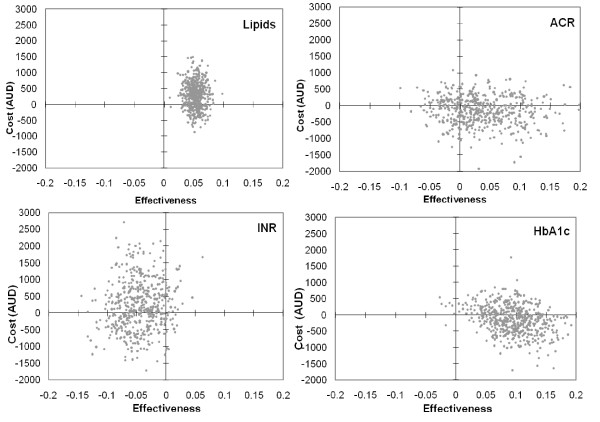
**Joint probability distribution of the incremental costs and effects of PoCT for INR, HbA1c, ACR and Lipids**.

The joint probability distribution of the incremental costs and incremental effectiveness was modelled for each PoCT strategy. The results are shown in Figure 1.

The joint probability distribution for INR is rather diffuse, with all but a small proportion lying either within the north-west (55.2%) or south-west (37.4%) quadrants of the ICER plane. This suggests that the main problem for this test is that it is less effective than a strategy based on laboratory testing. For HbA1c, virtually all the joint probability distribution lies within the north-east (36.4%) or south-east (62.2%) quadrants. For ACR testing, 47.4% of the bootstrapped results lie in the dominant quadrant (south-east quadrant) and 27.6% of the distribution lies in the north-west or south-west quadrants. For lipid testing, the majority of the joint probability distribution lies in the north-east quadrant (71.8%), indicating a trade-off between greater effectiveness and higher cost. Because the intermediate outcome indicator was specific to this study and not readily comparable with other interventions, it is unclear as to how much societal decision-makers would be willing to pay for maintaining an additional patient in therapeutic range.

## Discussion

This aspect of the Trial focused on evaluating the costs per patient and the incremental cost-effectiveness of implementing a PoCT strategy in GP compared to laboratory testing for INR, HbA1c, ACR and lipids within a time horizon of 18 months. For all tests, there was no statistically significant difference in overall costs between point of care and laboratory testing.

The study found a decrease in costs for hospital admissions for PoCT for HbA1c, INR and ACR, although none were statistically significant. This may have resulted from improved management of patients when PoCT is used as demonstrated in our Trial [9], which may in turn have resulted in reduced hospital visits for patients with diabetes or on anticoagulant therapy. In contrast, hospital admission costs increased for the lipids PoCT group, but again were not statistically significant. The sensitivity analyses indicated that, across all tests, the costs of hospital admissions were the most uncertain. It is likely that these results reflect the low power of the study to detect differences in hospitalisation.

For HbA1c and ACR, the point estimates of the costs of the PoC testing strategy were higher than in the control group. This appeared to be largely due to the establishment costs associated with introducing PoCT into a practice, the estimation of which required assumptions such as the useful clinical life and the relevant volume of testing. The establishment costs included the cost of the device, training, and quality assurance and represent a key investment for the general practitioner, particularly as a single general practice will never obtain the economies of scale and scope found in a pathology laboratory. This element of the cost per patient would decrease if patient volume increases.

Across the Trial, all PoC testing resulted in an increase in the number of tests per person-year when compared to the control group, but the greatest increase was found for INR (12.0 tests per-person year compared with 9.3 tests per person-year in the controls). Clinical guidelines recommend INR testing every 4-6 weeks [11], whereas testing for HbA1c [12] and ACR [12,13] is recommended every six months and 12 months respectively.

With PoCT, INR resulted in higher consultation costs and this may relate to the higher number of GP visits per person-year for the intervention group (19.2 visits per person-year compared to 13.6 visits per person-year in the control group). This in turn may result from the Trial protocol, which required PoCT to be undertaken in conjunction with a GP consultation.

With lipid testing, higher costs for pharmaceuticals were found for the intervention group. This may again relate to the better management of patients as shown by Bubner et al [9], more regular testing (within the guidelines) and greater adherence to medications, resulting in higher volumes of drugs being prescribed. The Trial found the same or better adherence to medication for hyperlipidaemia patients in the PoCT group compared with the control group [14].

These incremental costs per patient were most sensitive to differences in the costs of hospitalisation, and somewhat sensitive to differences in the costs of GP consultations, pharmaceuticals and allied health visits.

All four point of care tests led to significant savings for patients and their families, (travel costs and copayments) and, with the exception of INR, significant reductions in time seeking healthcare. However, these costs were not of sufficient magnitude to have much influence on the overall results (less than 5% of overall costs). To some extent our results concur with other reports. Jowett et al [15], in studying primary and secondary care anticoagulation clinics in six countries, found that costs to the patients were considerable, with the main driver being time costs. Parry et al [16] found that travel time and time spent in the clinic were significantly lower for patients attending primary care clinics rather than secondary care clinics for anticoagulant management. From a patient perspective, PoCT can allow patients to have a pathology test and see the GP about the results in one visit and thus can lead to significant savings.

It is noteworthy that 91% of patients in the Trial held health care cards or were pensioners, with the majority of practices being located in lower socio-economic status areas. This meant that most Medicare items used in the Trial were bulk-billed so that patients did not make a co-payment.

### INR

The results of the base case analysis found INR to be both less effective and more costly, and this was supported by the sensitivity analyses. Most of the joint probability distribution for INR was in the north-west or south-west quadrants. This is strong evidence that the INR PoCT strategy implemented in the Trial was not cost-effective, at least over the duration of follow-up in this study.

### HbA1c

The results of the cost-effectiveness analysis for HbA1c placed virtually all of the joint probability distribution within the north-east (trade-off) or south-east (dominant) quadrants. Although this result is somewhat favourable, any recommendation to implement this test at the point of care would depend on the value society would place on maintaining a patient within the therapeutic range.

### ACR

Although the point estimate of the ICER for ACR was in the dominant quadrant, the joint probability distribution was somewhat diffuse, extending into all four quadrants and thus suggesting that before a positive policy recommendation can be made, more precision in the estimate of the ICER is required.

### Lipids

For lipid testing, the point estimate of the ICER and most of its joint distribution are within the north-east quadrant, indicating a trade-off between incremental costs and incremental effectiveness. As the incremental effectiveness is not expressed in life-years gained or QALYs, it is uncertain whether the trade-off involved would be acceptable to the policy maker. Thus, the implementation of these tests using point of care would depend on the value society would place on maintaining a patient within the therapeutic range.

Comparing the results of the cost and the cost-effectiveness analyses for this Trial with other studies is difficult because of the limited research in this area [16-22]. Those studies that do exist are also limited in their scope and design. Only one study directly compared GP PoCT with usual care using laboratory testing [21]. Moreover, while some studies investigated cost-effectiveness, [16,19,22] only one reported the incremental cost-effectiveness ratio [19].

The Trial showed the point estimate of the INR PoCT strategy to be dominated by laboratory testing. This contrasts with the results of a Belgian study which found the use of a PoCT device for anticoagulant management combined with a multifaceted education intervention was dominant to usual care in GP [19]. Anticoagulant management in GP using PoCT, was also found to be more costly by Parry et al [22]. This UK study investigated the cost-effectiveness of PoCT, using data obtained through an RCT of PoCT and computerised decision support to manage anticoagulation therapy in GP. It found that the costs per patient-year were significantly higher in primary care than in hospital-based clinics because of the less efficient use of fixed costs. While sensitivity analysis showed that costs to primary care could be reduced, they still remained higher than secondary care. The cost drivers were clinic sizes and the number of visits. The reduced cost to patients and their families using PoCT for anticoagulant management in this study confirmed a previous finding by Parry et al [16]. In contrast, another study which investigated HbA1c PoCT in a GP setting found no differences in patient-borne costs between the intervention and control group (laboratory testing) [21].

The results for HbA1c from this Trial are congruent with work undertaken in the United Kingdom by Khunti et al [21]. This small study assessed the effect and cost of using PoCT for HbA1c testing in the management of Type 2 diabetes in GP. They found no difference in the total cost for diabetes care using PoCT compared with laboratory testing.

There are no previous studies about the comparative costs and cost-effectiveness of PoCT for ACR or lipids in a GP setting.

### Limitations

This cost-effectiveness analysis had a number of limitations. Firstly, the Trial used an intermediate outcome indicator in the ICER. This indicator is specific to this Trial and not immediately generalisable, unlike life-years gained or QALYs, thus making the interpretation of the acceptability of the trade-offs difficult. Additionally, the short duration of the Trial may have limited the identification of any clear health gains, which in turn would influence the ICER.

Secondly, limitations arose from using Medicare data to estimate costs and volumes. Medicare data were used to determine the volume of tests used in the costing analysis, because analysis of the data provided directly from the practices and Pathology Providers indicated under-reporting of results, particularly for the control group. However, with Medicare data, the number of tests claimed is not identifiable by type of medical practitioner, and so some tests included in the analysis may have been ordered for that patient by a specialist and not a general practitioner.

Thirdly, the wide ranges of the CIs suggest that the Trial may have been underpowered for measuring costs. The power calculation for the Trial was based on the primary outcome of the proportion of patients with test results within the target range. Fourthly, Pharmaceutical Benefits Scheme (PBS) data provides incomplete drug utilisation history, as only scripts priced over the copayment threshold are recorded. However, as the PBS was used for both arms of the Trial, this limitation would apply to both groups.

Finally, the PoCT Trial was not able to recruit sufficient patients in two of the three conditions to obtain the desired power of 80%, which may limit the interpretation of the results.

## Conclusions

The results add to the small body of work on the economic value of PoCT. To obtain the maximum available health benefits from scarce clinical resources, it is important that new health technologies be assessed for their cost-effectiveness. Health care funders are increasingly relying on information to assist them in maintaining value for money. This should also minimise waste of these resources. In this Trial, the cost-effectiveness for PoCT with ACR was found to be somewhat favourable, thereby providing evidence to support its implementation in GP in Australia. On the other hand, INR by PoCT was not cost-effective and its introduction into routine GP is not supported by this study. This suggests that further research into the technology itself might be worth pursing or into a better regime of testing. Moreover, substantial uncertainty still surrounds PoCT for HbA1c and Lipids, suggesting more development work before PoCT can be introduced into GP.

Cost-effectiveness analysis is merely one useful tool for the decision-maker. While the results of this study provide valuable information about the effectiveness and costs for PoCT in GP, other factors need to be considered when determining whether this strategy should be implemented nation-wide [23]. These include GP, device operator and patient satisfaction with PoCT, its impact on GP management and improved adherence to medications by intervention patients. The PoCT Trial has demonstrated that: patients are satisfied with PoCT and find it acceptable [24]; that PoCT provides an effective alternative to pathology laboratory testing and can enhance GP management of patients with chronic disease [9]; and that PoCT can assist patients in self-management behaviours such as medication adherence [14]. The costs associated with introducing PoCT within a quality framework were high for the Trial and involved accreditation, a quality assurance program and training. While a quality framework is essential for the introduction of PoCT in GP, it may be possible to achieve the same quality outcomes at a reduced cost by adding these to existing organisations or programs or by using alternatives such as online training. These factors may influence the decision to support PoCT.

## Competing interests

The authors declare that they have no competing interests.

## Authors' contributions

CL participated in the design and coordination of the study, compilation of data, performed some analysis and drafted the manuscript. JM participated in the design and coordination of the study, oversaw the analysis, contributed to the interpretation of the results and critically revised the paper. NB performed the statistical analysis and drafted the manuscript. JB contributed to the design of the study and critically revised the paper. All authors read and approved the final version of the manuscript.

## Pre-publication history

The pre-publication history for this paper can be accessed here:

http://www.biomedcentral.com/1472-6963/10/165/prepub

## Supplementary Material

Additional file 1 **Comparison of direct and indirect costs at 18 months for INR, HbA1c, ACR and Lipid tests - costs per patient (Australian dollars, calendar year 2006)**. This file provides a comparison of costs (direct and indirect) for the other tests investigated, namely INR, ACR and Lipids. Click here for file

Additional file 2 **One-way sensitivity analysis for the difference in direct health care sector costs per patient for PoC INR, HbA1c, ACR and Lipid testing compared to a laboratory INR, HbA1c, ACR and Lipid testing**. This file provides the one-way sensitivity analysis for the difference in direct health care sector costs per patient for PoC testing compared to laboratory testing for INR, HbA1c, ACR and Lipids Click here for file
