# Supplementary material for: The cost-effectiveness of point of care testing in a general practice setting: results from a randomised controlled trial
Source: BMC Health Serv Res. 2010 Jun 15;10:165. doi: 10.1186/1472-6963-10-165 (PMC2905350; doi:10.1186/1472-6963-10-165)
Supplement: Additional file 1 — Comparison of direct and indirect costs at 18 months for INR, HbA1c, ACR and Lipid tests - costs per patient (Australian dollars, calendar year 2006). This file provides a comparison of costs (direct and indirect) for the other tests investigated, namely INR, ACR and Lipids. [file 1472-6963-10-165-S1.DOC]

Additional File 1

Comparison of direct and indirect costs at 18 months for INR tests – costs per patient (Australian dollars, calendar year 2006)

| **RESOURCES** | **INTERVENTION (PoCT)**  **N=572** | **CONTROL (Laboratory)**  **N=372** | **DIFFERENCE (INTERVENTION – CONTROL)** |
| --- | --- | --- | --- |
| **Direct Costs to the health care sector** | **(95% CI)** | **(95% CI)** | **(95% CI)** |
| Establishment costs in GP | $121 | $0* | $121 |
| Consumables & maintenance in GP | $107 ($77, $137) | $0* | $107 ($77, $137) |
| Quality assurance & control in GP | $47 | $0* | $47 |
| INR tests (100% MBS fee in GP, 85% MBS fee in pathology laboratory) | $326 ($221, $359) | $565 ($383, $621) | -$239 (-$310, -$165) |
| **Sub total cost of actual test** | **$606 ($416, $634)** | **$565 ($383, $621)** | **$36 (-$54, $120)** |
| GP consultations | $938 ($848, $1,049) | $708 ($632 , $772) | $229 ($120, $367) |
| Hospital admissions | $1,048 ($214, $2,060) | $1,174 (-$37, $2,019) | -$126 (-$1,343, $1,568) |
| Emergency Dept visits | $19 ($4 , $34) | $14 ($5, $22) | $4 (-$12, $24) |
| Specialist consultations | $188 ($171, $204) | $198 ($183, $212) | -$10 (-$31, $11) |
| Allied health visits | $251 ($220, $285) | $250 ($203, $301) | $1 (-$61, $66) |
| Pharmaceuticals | $131 ($124, $138) | $113 ($101, $124) | $18 ($6, $30) |
| **Subtotal direct costs to healthcare sector** | **$3,175 ($2,163, $4,092)** | **$3,023 ($1,669, $3,730)** | **$153 (-$1,097, $1,828)** |
| **Direct Costs to the patients and families** |  |  |  |
| Copayment for GP consultations and pharmaceuticals | $4 ($4, $5) | $4 ($2, $7) | $0 (-$2, $3) |
| Patient travel costs | $20 ($10, $24) | $24 ($18, $26) | -$4 (-$15, -$2) |
| **Subtotal direct costs to patients and families** | $24 ($14, $38) | $28 ($23, $31) | -$4 (-$16, -$3) |
| **Indirect Costs** |  |  |  |
| **Time seeking healthcare** | **$97 ($86, $100)** | **$99 ($89, $107)** | **-$2 (-$16, $5)** |
| **Total costs (both sectors)** |  |  |  |
| **Total** | **$3,297 ($2,262, $4,197)** | **$3,150 ($1,786, $3,853)** | **$147 (-$1114, $1816)** |
| *Note: totals not exact due to rounding*  **For the control group these items are included in the MBS fee for the laboratory* | | | |

**Comparison of costs (direct and indirect) at 18 months for Urine Albumin Creatinine (ACR) tests - costs per patient (Australian dollar, calendar year 2006)**

| **RESOURCES** | **INTERVENTION (PoCT)**  **N=1182** | **CONTROL (Laboratory)**  **N=785** | **DIFFERENCE (INTERVENTION – CONTROL)** |
| --- | --- | --- | --- |
| **Direct Costs to the health care sector** | **(95% CI)** | **(95% CI)** | **(95% CI)** |
| Establishment costs in GP | $87 | $0* | $87 |
| Consumables & maintenance in GP | $20 ($14, $22) | $0* | $20 ($14, $22) |
| Quality assurance & control in GP | $18 | $0* | $18 |
| Microalbumin tests (100% MBS fee in GP, 85% MBS fee in pathology laboratory) | $43 ($35, $47) | $57 ($48, $63) | -$14 (-$24, -$5) |
| **Sub total cost of actual test** | **$168 ($132, $214)** | **$57 ($48, $63)** | **$111 ($76, $157)** |
| GP consultations | $579 ($515, $659) | $560 ($502, $618) | $18 (-$69, $112) |
| Hospital admissions | $171 (-$181, $662) | $506 (-$137, $1,241) | -$334 (-$1,131, $460) |
| Emergency Dept visits | $8 ($3, $13) | $8 ($2, $13) | -$1 (-$8, $7) |
| Specialist consultations | $173 ($163, $183) | $169 ($157, $180) | $4 (-$11, $20) |
| Allied health visits | $221 ($195, $253) | $232 ($188, $282) | -$11 (-$72, $47) |
| Pharmaceuticals | $371 ($361, $462) | $363 ($351, $418) | $8 (-$9, $66) |
| **Subtotal direct costs to healthcare sector** | **$1,692 ($1,348, $2,265)** | **$1,895 ($1,268, $2,659)** | **-$204 (-$1,030, $641)** |
| **Direct Costs to the patients and families** |  |  |  |
| Copayment for GP consultations and pharmaceuticals | $5 ($5, $8) | $5 ($2, $8) | $0 (-$2, $5) |
| Patient travel costs | $12 ($10, $14) | $28 ($23, $33) | -$16 (-$22, -$11) |
| **Subtotal direct costs to patients and families** | **$17 ($16, $21)** | **$33 ($29, $40)** | **-$16 (-$22, -$10)** |
| **Indirect Costs** |  |  |  |
| **Time seeking healthcare** | **$18 ($16, $19)** | **$26 ($24, $28)** | **-$8 (-$14, -$8)** |
| **Total costs (both sectors)** |  |  |  |
| **Total** | **$1,727 ($1,387, $2,309)** | **$1,954 ($1,319, $2,712)** | **-$228 (-$1041, $625)** |
| *Note: totals not exact due to rounding*  **For the control group these items are included in the MBS fee for the laboratory* | | | |

Comparison of cost (direct and indirect) at 18 months for Lipid tests – costs per patient (Australian dollars, calendar year 2006)

| **RESOURCES** | **INTERVENTION (PoCT)**  **N=2536** | **CONTROL (Laboratory)**  **N=1463** | **DIFFERENCE (INTERVENTION – CONTROL)** |
| --- | --- | --- | --- |
| **Direct Costs to the health care sector** | **(95% CI)** | **(95% CI)** | **(95% CI)** |
| Establishment costs in GP | $23 | $0* | $23 |
| Consumables & maintenance in GP | $38 ($21, $43) | $0* | $38 ($21, $43) |
| Quality assurance & control in GP | $26 | $0* | $26 |
| Lipid tests (100% MBS fee in GP, 85% MBS fee in pathology laboratory) | $54 ($36, $59) | $78 ($63, $78) | -$24 (-$37, -$18) |
| **Sub total cost of actual test** | **$141 ($103, $143)** | **$78 ($63, $77)** | **$63 ($32, $71)** |
| GP consultations | $573 ($509, $651) | $503 ($457, $544) | $70 (-$7, $155) |
| Hospital admissions | $417 (-$178, $1,098) | $276 (-$139, $692) | $142 (-$554, $985) |
| Emergency Dept visits | $2 ($0, $10) | $0 ($0, $12) | $1 (-$6, $5) |
| Specialist consultations | $166 ($157, $176) | $168 ($158, $179) | -$2 (-$16, $13) |
| Allied health visits | $196 ($166, $227) | $175 ($169, $249) | $20 (-$63, $40) |
| Pharmaceuticals | $1190 ($987, $1,268) | $938 ($905, $1,108) | $252 ($143, $296) |
| **Subtotal direct costs to healthcare sector** | **$2,686 ($1,953, $3,199)** | **$2,139 ($1,814, $2,702)** | **$547 (-$468, $1096)** |
| **Direct Costs to the patients and families** |  |  |  |
| Copayment for GP consultations and pharmaceuticals | $7 ($6, $9) | $7 ($2, $8) | $0 (-$1, $6) |
| Patient travel costs | $17 ($11, $18) | $24 ($20, $28) | -$7 (-$15, -$7) |
| **Subtotal direct costs to patients and families** | $23 ($18, $24) | $30 ($27, $36) | -$7 (-$16, -$6) |
| **Indirect Costs** |  |  |  |
| **Time seeking healthcare** | **$24 ($20, $26)** | **$33 ($31, $35)** | **-$9 (-$14, -$9)** |
| **Total costs (both sectors)** |  |  |  |
| **Total** | **$2,732 ($1,994, $3,241)** | **$2,202 ($1,875, $2,765)** | **$530 (-$489, $1,078)** |
| *Note: totals not exact due to rounding*  **For the control group these items are included in the MBS fee for the laboratory* | | | |
