# Supplementary material for: The cost-effectiveness of point of care testing in a general practice setting: results from a randomised controlled trial
Source: BMC Health Serv Res. 2010 Jun 15;10:165. doi: 10.1186/1472-6963-10-165 (PMC2905350; doi:10.1186/1472-6963-10-165)

Additional file 2

One way sensitivity analysis for the difference in direct health care sector costs per patient for PoC INR testing compared to a laboratory INR testing


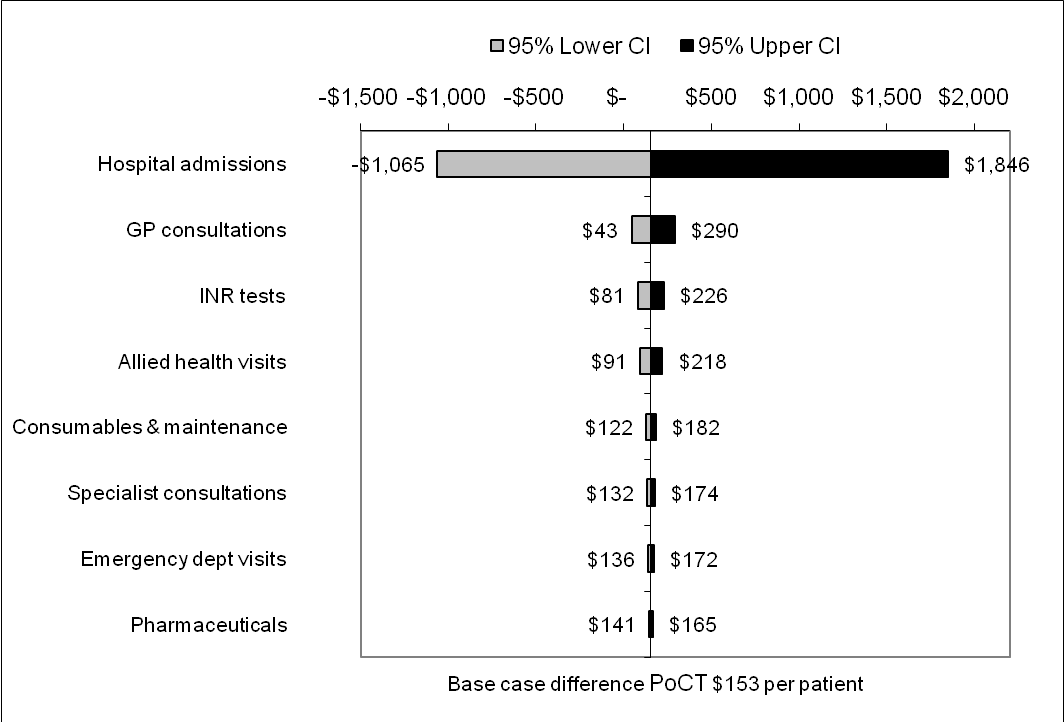


One way sensitivity analysis for the difference in direct health care sector costs per patient for PoC HbA1c testing compared to a laboratory HbA1c testing


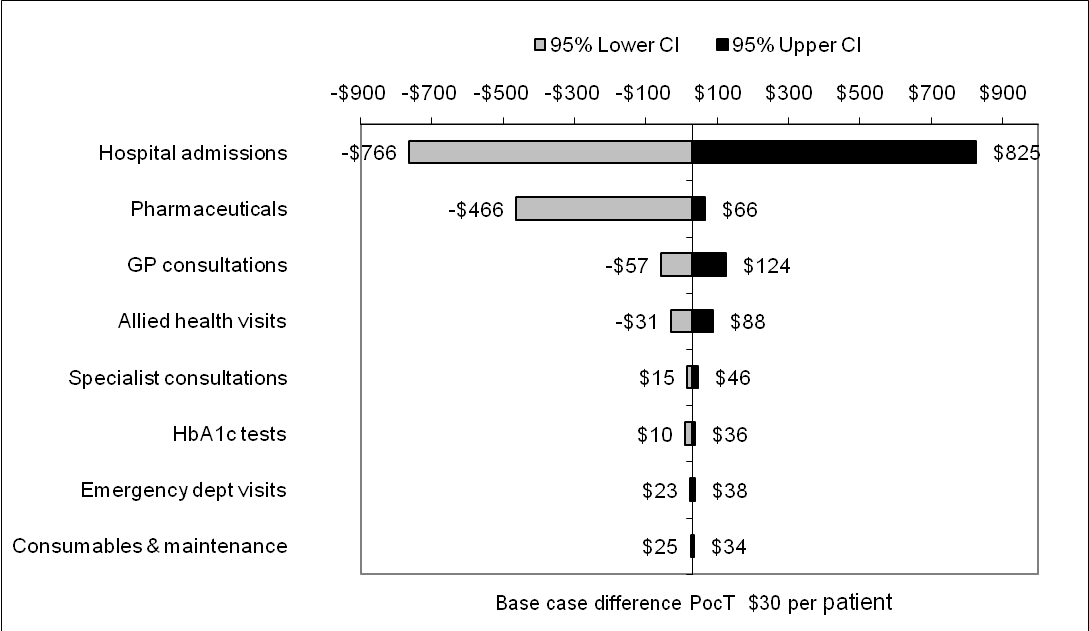


One way sensitivity analysis for the difference in direct health care sector costs per patient for PoC ACR testing compared to a laboratory ACR testing


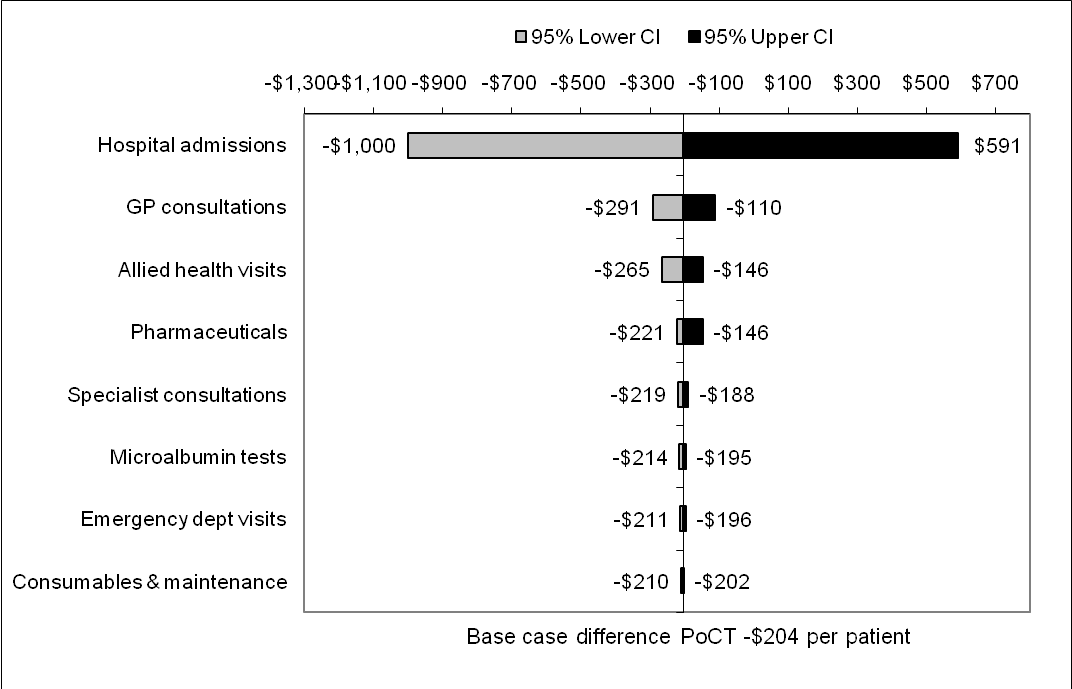


One way sensitivity analysis for the difference in direct health care sector costs per patient for PoC Lipid testing compared to a laboratory Lipid testing


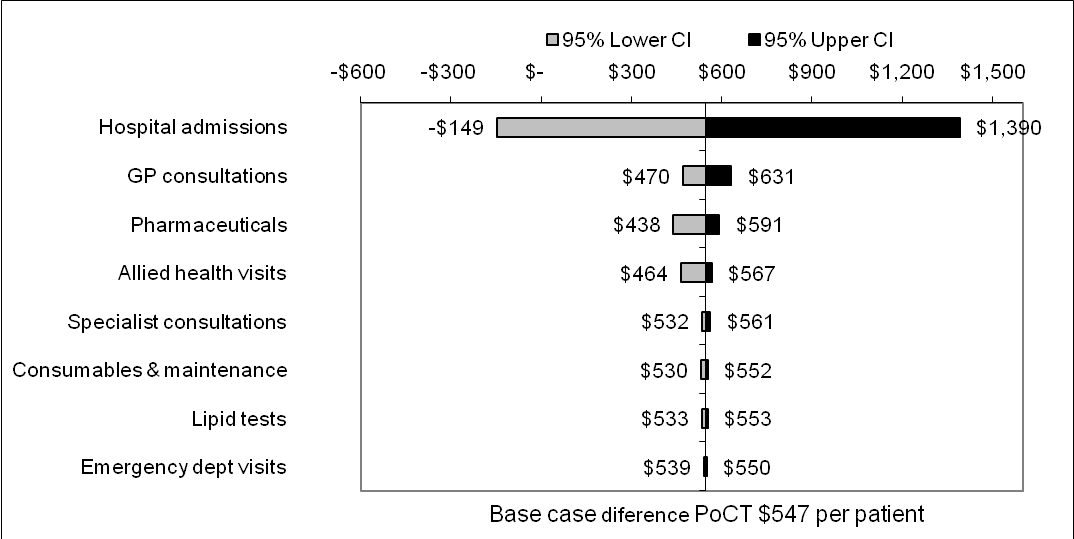

Supplement: Additional file 2 — One-way sensitivity analysis for the difference in direct health care sector costs per patient for PoC INR, HbA1c, ACR and Lipid testing compared to a laboratory INR, HbA1c, ACR and Lipid testing. This file provides the one-way sensitivity analysis for the difference in direct health care sector costs per patient for PoC testing compared to laboratory testing for INR, HbA1c, ACR and Lipids [file 1472-6963-10-165-S2.DOC]
